# Single cone versus carrier-based obturation using a premixed bioceramic sealer in postgraduate master cohort: a 36 months retrospective evaluation

**DOI:** 10.1007/s00784-026-06900-0

**Published:** 2026-05-11

**Authors:** Fausto Zamparini, Andrea Spinelli, Vincenzo Tosco, Maria Giovanna Gandolfi, Carlo Prati

**Affiliations:** 1 Department of Biomedical and Neuromotor Sciences, Endodontic Clinical Section, Dental School, University of Bologna, Bologna, Italy; 2 Department of Biomedical and Neuromotor Sciences, Laboratory of Green Biomaterials and Oral Pathology, Dental School, University of Bologna, Bologna, Italy; 3 Department of Life Sciences, Health and Health Professions, Link Campus University, Rome, Italy

**Keywords:** Calcium silicate sealers, Bioceramics, Extrusions, Retrospective studies, PAI

## Abstract

**Aim:**

To evaluate 36-month clinical outcome and occurrence of sealer apical extrusion events of teeth obturated with a premixed CaSi based sealer associated with a single cone technique or carrier-based technique.

**Methodology:**

In this retrospective cohort study, consecutive healthy patients were treated by a postgraduate master cohort. Teeth were shaped with NiTi rotary or reciprocating instruments, irrigated with 5% NaOCl and 10% EDTA, final irrigation with sterile water. Root canals were obturated with a premixed bioceramic-based sealer (NeoSealer Flo, Avalon Biomed, Houston, TX, USA) using either a warm carrier-based technique (Thermafil, Dentsply, Konstanz, Germany) or a cold single-cone technique. Patients were included in a recall program and teeth were examined at 3, 6, 24 and 36 months. Clinical and radiographic data were obtained and the following parameters were evaluated: preoperative Periapical Index (PAI) score and signs/symptoms, PAI score at follow‐up. Teeth were considered ‘healthy’ (PAI ≤ 2, no signs/symptoms) or with “endodontic lesion” (PAI ≥ 3, signs/symptoms present, retreated). Two calibrated examiners assessed outcomes blinded to preoperative status. Chi-square test evaluated the outcome between these two obturation techniques. Sealer Apical extrusion typologies and radiographic modifications were analysed through a computerized methodology on periapical radiographs.

**Results:**

A total of 53 root canal treatments (48 subjects, mean age 45.4 ± 12.1 years) with a minimum 36 months follow-up were analysed. Of these, 29 were filled with single cone technique, while 24 were obturated with a carrier-based technique. At 36-month evaluation, healing rate was 88% and 93% with regards to carrier-based or single cone technique with no statistical differences (P>0.050). Apical extrusion was observed in 18/24 teeth of patients filled with carrier-based technique (75%) and 14/29 in teeth with cold technique (48.2%). Mean extrusion size was 1.49 mm. Significantly higher extrusion (in mm) was observed in teeth with PAI ≥ 3 and in Carrier-based group. Radiographic modification of apically extruded sealer was observed in 5 of 32 extruded cases (15.6%).

**Conclusions:**

Clinical outcome shows a high rate of survived and healed teeth with both type of obturation techniques. Apical extrusion of the sealer can be frequent when using carrier-based technique or in presence of large diameters and periapical radiolucencies.

## Introduction

Hydraulic calcium silicate cements (CaSi) were originally introduced more than twenty years ago as root-end filling materials [1] and later adapted, through modifications in formulation and mechanical properties, for use as endodontic sealers [2, 3]. Their evolution from powder–liquid forms to premixed, flowable, “ready to use” materials has facilitated clinical handling and expanded their indications in everyday practice [2–4]. Premixed bioceramic sealers gained high popularity in clinical practice [3, 5, 6], exhibiting favorable physicochemical and biological properties, such as apatite nucleation ability, calcium release and alkalizing activity [7–9], good biocompatibility [10, 11], osteoconductive and osteoinductive potential [12, 13]. However, evidence supporting the superiority of these sealers in terms of postoperative pain reduction [13, 14] or long-term clinical outcomes remains limited, mainly due to the paucity of studies with follow-up periods exceeding 2–3 years [4].

**NeoSealer Flo** is a recently introduced premixed bioceramic sealer constituted by tricalcium silicate (< 25%) and dicalcium silicate (< 10%) as bioactive components, and calcium aluminate (< 25%), calcium aluminum oxide (grossite) (< 6%), tricalcium aluminate (< 5%) and tantalite (50%) as radiopacifier, and traces of calcium sulfate (< 1%) [8]. According to the manufacturer instructions, Neosealer Flo was designed for application in both warm and cold single cone techniques; however, no studies evaluating its use in combination with carrier-based obturation techniques have been reported in the literature [15].

Carrier-based techniques offer documented advantages, including improved gutta-percha adaptation, a reduced operator learning curve, and reliable clinical outcomes. These aspects are particularly relevant in educational settings, where treatments are performed by postgraduate students. Furthermore, warm procedures have been traditionally paired with epoxy resin–based sealers [16–18]; their combination with hydraulic bioceramic sealers is less documented, and some concerns from in vitro investigations persist regarding the influence of temperature on sealer setting time, flowability, and film thickness of calcium silicate sealers, potentially affecting obturation quality [19–22].

Another clinically relevant aspect to be considered is sealer extrusion beyond the apical foramen, which represents a relatively frequent and undesired event following root canal obturation. To date, evidence regarding the impact of sealer extrusion on periapical healing remains inconclusive [23, 24].

Interestingly, radiographic modifications of extruded material have been reported for several premixed hydraulic sealers, in contrast to traditional epoxy resin–based sealers, which typically remain radiographically stable and confined close to the apical region over time [4].

No clinical studies have specifically evaluated the performance of NeoSealer Flo in carrier-based techniques, and little information is available on extrusion frequency, radiographic evolution, or periapical healing patterns.

Therefore, this retrospective cohort study aims to evaluate the 36-month clinical and radiographic outcomes of root canal treatments using the bioceramic sealer NeoSealer Flo in combination with either carrier-based or cold single-cone obturation techniques. The null hypotheses tested were:

 [1] that no significant differences would be observed between carrier-based and cold single-cone obturation techniques in terms of clinical success and periapical healing outcomes when using NeoSealer Flo;

 [2] that the obturation technique would not influence sealer extrusion frequency or the radiographic behavior of the extruded material over time.

## Materials and methods

### Study design and sample

This retrospective cohort study included consecutive healthy patients treated at the Endodontic Clinical Section between 2020 and 2022. All procedures were performed by postgraduate master operators (*n* = 9 operators). Before the start of the study, the operators performed a training course in the clinical use of either the warm carrier-based technique with NeoSealer Flo or the single-cone technique with NeoSealer Flo. The obturation technique was selected according to operator preference. All treatments were performed under strict supervision by experienced endodontic tutors following a standardized clinical protocol [16, 25, 26]. The study was designed in accordance with the STROBE [27] guidelines for observational studies.

All patients were treated in compliance with the ethical standards established in the Declaration of Helsinki, as revised in 2013 [28]. Written informed consent was obtained from all participants prior to treatment and data collection. The local ethics committee approved the study protocol (OUTENDO 2020, protocol 461/2020/OSS/AUSLBO).

### Inclusion and exclusion criteria

Tables 1 and 2 reports in detail the inclusion and exclusion criteria of the study.

**Table 1:** Inclusion criteria

1. Age 18–75 years
2. ASA 1–2
3. At least one tooth affected by endodontic pathology (pulpitis, pulp necrosis, recurrent lesions after previous endodontic treatment)

**Table 2:** Exclusion criteria

1. Teeth with fewer than 2 intact crown walls
2. Teeth used as abutments for fixed restorations
3. Active periodontal disease (PPD > 4 mm, BoP > 25% of sites)
4. Wide apices or radiographic obscuration of the pulp chamber
5. Presence of systemic disease that may compromise bone healing or the immune response
6. Pregnancy or breastfeeding
7. Smoking (> 15 cigarettes/day)
8. Exposure to radiation therapy to the head and neck region or malignant disease involving the jaws
9. Absence of occlusal contacts

**Table 3:** Patient related characteristics between the two obturation groups

Patient level	Single cone + Neosealer Flo *n* = 26	Carrier-based + Neosealer Flo *n* = 22	Chi- square
*Sex*			
Male	18 (69.2)	14 (63.6)	Chi = 0.168
Female	8 (30.8)	8 (36.4)	*p* = 0.682
*Age*,* Years*			
18–30	7 (26.9)	3 (13.6)	Chi = 1.42
31–65	11 (42.3)	12 (54.6)	*p* = 0.493
> 65	8 (30.8)	7 (31.8)	

**Fig. 1:**
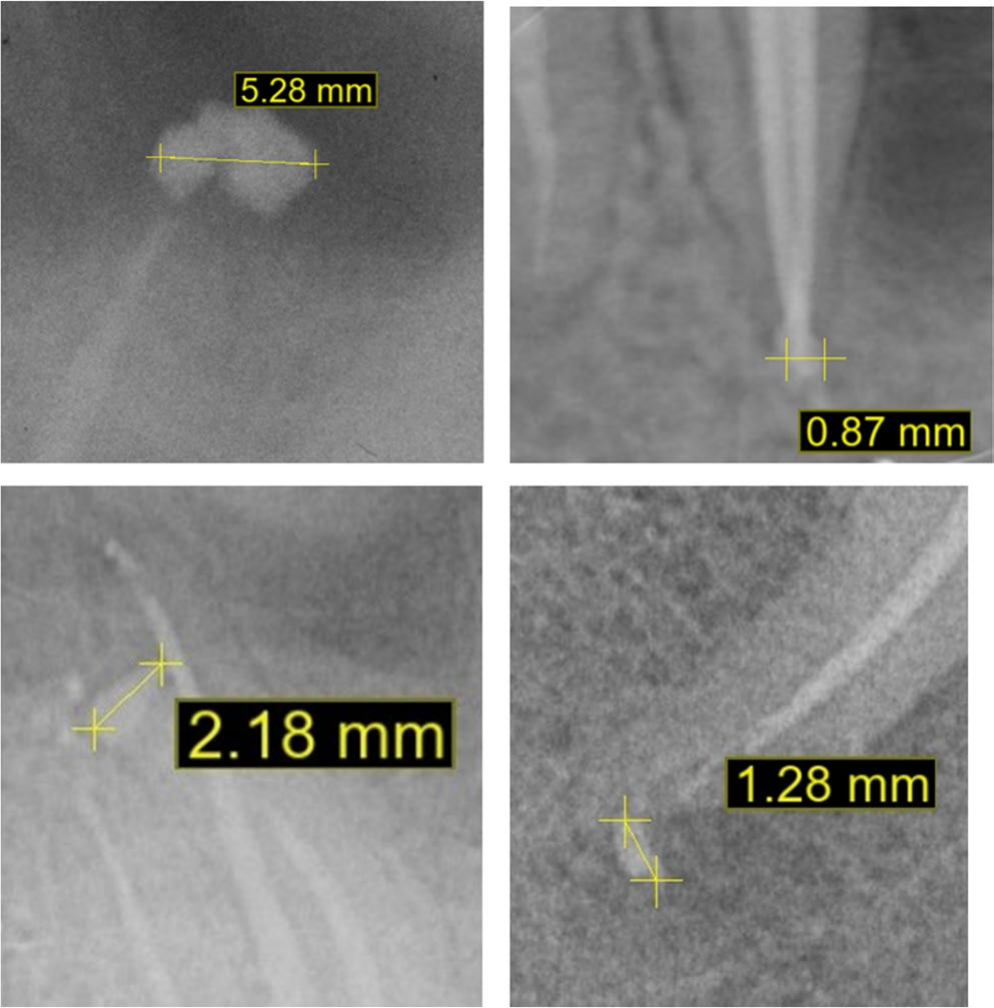
Examples of apical extrusion measurements using the digital software. The images revealed extrusions of different size (from 0.87 mm to over 5 mm) and shapes (linear, round shaped and irregular shaped morphologies), due to the presence or absence of periapical lesions

**Fig. 2:**
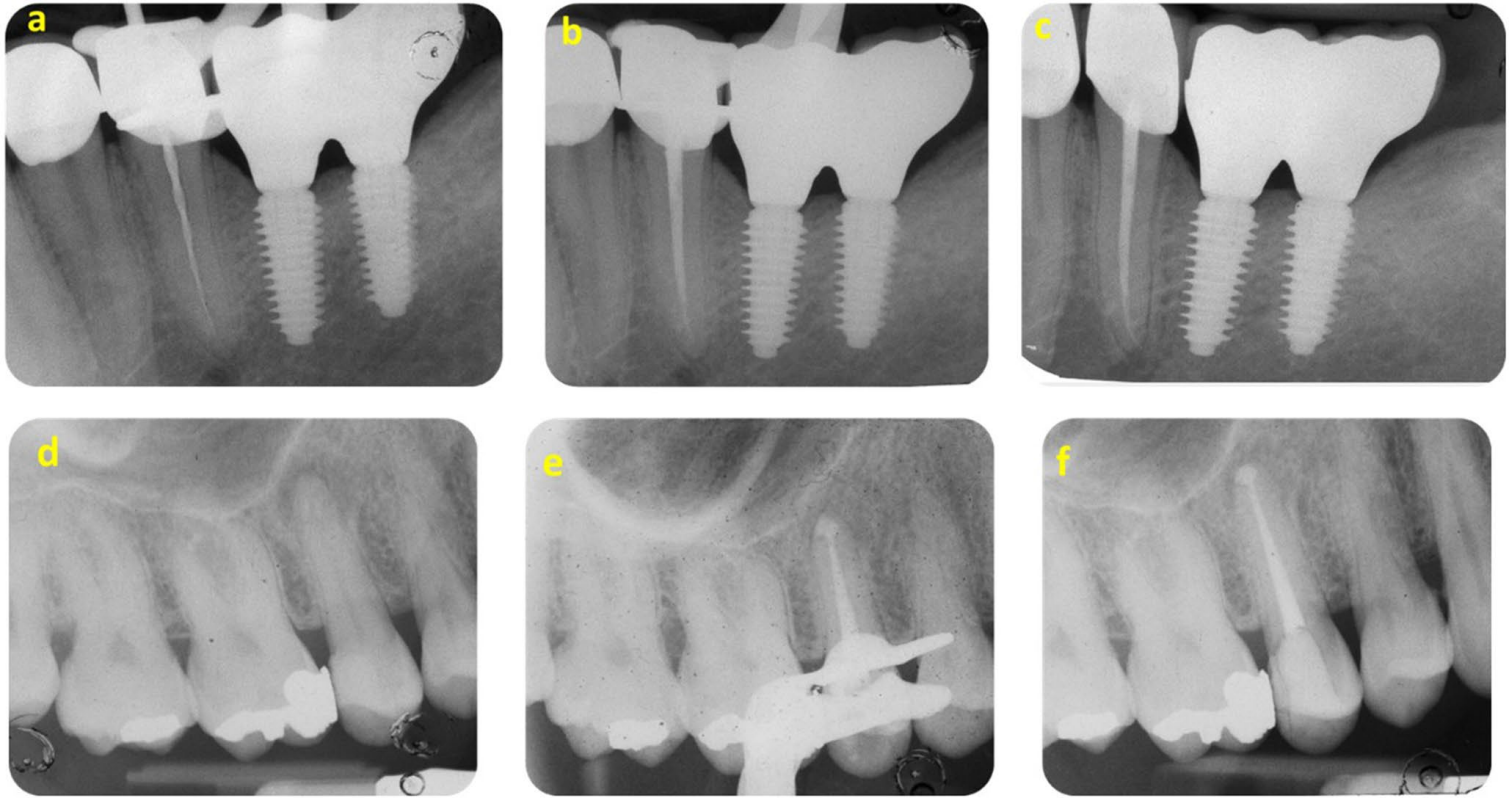
Representative cases of Single cone + Neosealer Flo, and Carrier-based + Neosealer Flo. Case 1 (a-c) pulpitis case (PAI 2), in a tooth with a full Zirconia crown. X-ray at Working Length, root canal filling with single cone technique and NeoSealer Flo. Sealer remained stable and healthy tissues were observed at the follow-up. Case 2 (d-f) apical lesion (PAI 4) on asymptomatic upper premolar. The premolar presented a deep composite reconstruction. X ray after filling with carrier-based technique and NeoSealer Flo. Apical extrusion was observed but remained stable up to the final follow-up. No Apical re-exacerbation was observed

**Fig. 3:**
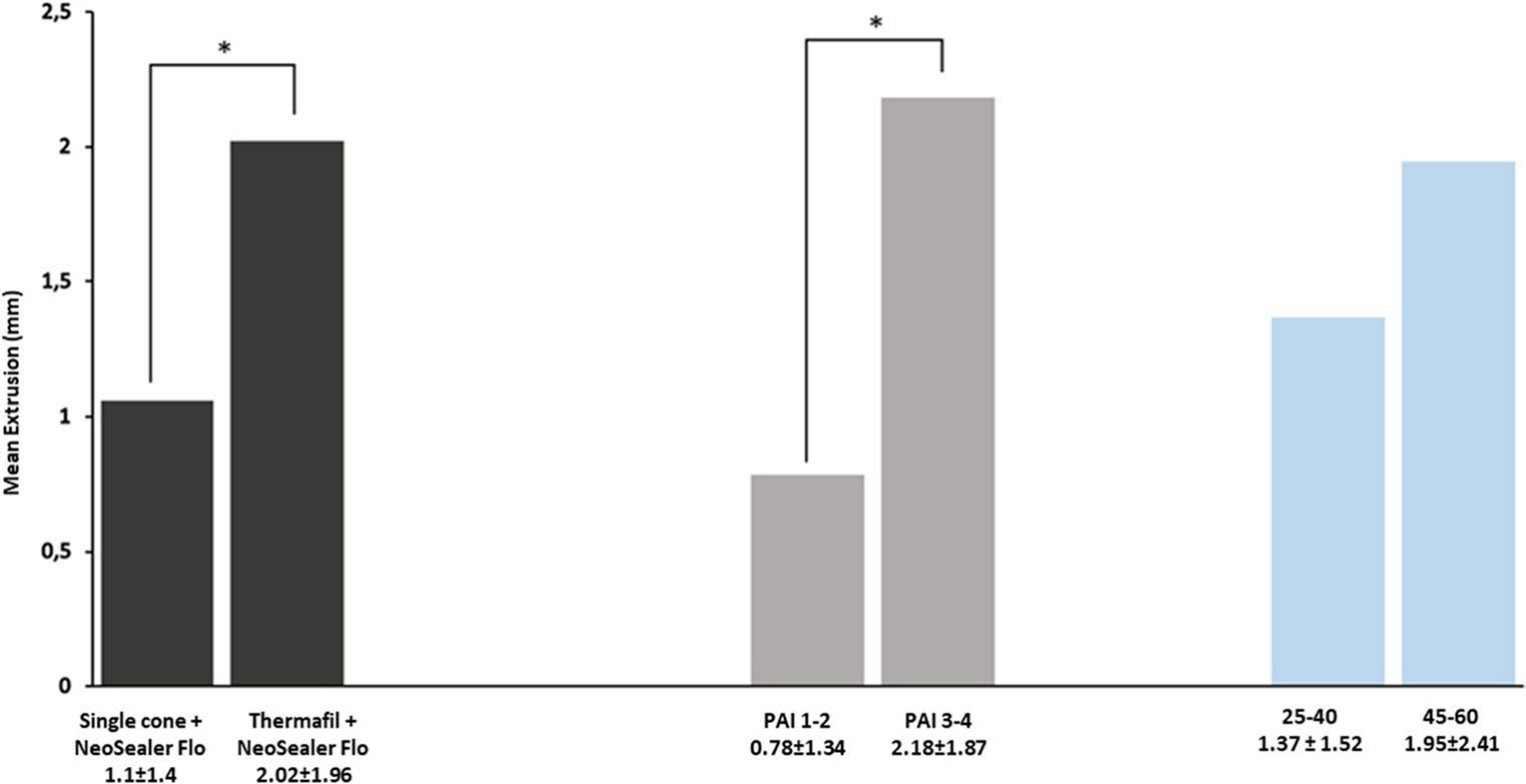
Extrusion comparisons according to obturation technique, initial PAI and final apical diameter. Asterisks indicate a statistically significant difference between parameters (*p*<0.050). Teeth that were filled with carrier-based technique (Carrier-based +NeoSealer Flo), or presented a periapical lesion (PAI 3–4) at baseline had significantly higher extrusion diameters (*p*<0.050). teeth with larger apical diameters (size 45–60) had higher extrusion diameters, but not significantly different from teeth with normal apical diameter (size 25–40) (*p*>0.050)

### Root canal treatment procedures

Root canal treatments were conducted under local anesthesia (Carboplyne 30 mg/ml, Dentsply, Germany) and rubber dam isolation. Working length was determined using a combination of electronic apex locator (Root ZX, Morita Europe, Dietzenbach, Germany) and periapical radiographs. Root canals were shaped using either NiTi rotary systems (primary treatments) or reciprocating instruments (non-surgical retreatments).

Canals were irrigated using 5% sodium hypochlorite (NaOCl) and 10% ethylenediaminetetraacetic acid (EDTA). Final irrigation was performed with 2 mL of sterile water before obturation.

### Root canal filling techniques and group allocation 

All root canals were obturated using a bioceramic-based premixed sealer (NeoSealer Flo, Avalon Biomed, Houston, TX, USA), in combination with either a carrier-based technique (Thermafil, Dentsply, Konstanz, Germany) or a single cone cold technique. In the carrier-based technique group, obturation was performed using a pre-heated carrier-based system. In the cold technique group, the canal was filled with a single gutta-percha cone matched to the master apical file.

The root canal filling treatments were performed by 2 different operator groups previously trained for only one technique. Post-graduate operators (*n* = 9) of the Master Program in Endodontics were assigned to receive a full training course on the clinical use of the carrier-based technique with NeoSealer Flo or the use of cold single cone technique with NeoSealer Flo. Sealer application was done with a K-file to working length − 3 mm. The excess gutta-percha in warm techniques was removed with a heated instrument. After that, interim provisional restoration was performed using Coltosol and a cotton pellet [29]. Post-endodontic reconstruction protocols did not differ between the groups. After approx. 2 weeks, Coltosol temporary restoration was removed and crown was restored using self-etching dentinal bonding agent (Clearfil Se Bond, Kuraray, Osaka, Japan), photocured for 30 s and layered using a flowable composite resins (G-Aenial, GC Corporation, Tokyo, Japan).

### Follow-up and clinical evaluation

Patients were re-evaluated up to 36 months after treatment. Follow-up included clinical examination and periapical radiographs using standardized paralleling technique. Radiographs were scanned at 1,000 dpi resolution with ×20 magnification and analyzed using ImageJ software (NIH, Bethesda, MD, USA).

### Radiographic assessment

Preoperative and follow-up radiographs were scored using the Periapical Index (PAI) [30]. Two calibrated and blinded examiners independently assessed radiographs. In cases of disagreement, a consensus was reached. Calibration was confirmed with a weighted kappa of 0.90 for both inter- and intra-examiner agreement.

Sealer extrusion was recorded and measured on each periapical radiograph using the “cephalometric analysis” section of the BlueSkyPlan software (Blue Sky Bio, Libertyville, USA). A reference was set 1 mm above the x-ray film and the software was calibrated taking this measurement into account. Using the “ruler” tool, the long axis of the sealer extruded beyond the apex was measured very accurately (Fig. 1a, b).

### Definition of success and survival criteria

Teeth were classified according to the following criteria:

#### Healthy

PAI ≤ 2 and absence of clinical signs or symptoms.

#### Endodontic lesion

PAI ≥ 3 and/or presence of clinical signs or symptoms (pain, swelling, sinus tract), or need for retreatment.

These categories were used to define treatment outcomes. The **success rate** included only teeth classified as healthy, whereas the **survival rate** included both healthy and endodontic lesion groups [16, 25, 26].

### Statistical analysis

Categorical variables were expressed as counts and percentages and compared between the two obturation groups (carrier-based vs. single-cone groups) using the Chi-square test. Descriptive statistics were used to report the distribution of sealer extrusion types and radiographic changes. Continuous variables were summarized as mean ± standard deviation and median [interquartile range] and analysed through t-test. Healing rates were reported with 95% confidence intervals, and the absolute difference in healing rates between groups was calculated with its 95% confidence interval. A two-sided p-value < 0.05 was considered statistically significant. As this was a retrospective study including all consecutive eligible cases treated during the study period and available at a minimum 36-month follow-up, no a priori sample size calculation was performed. No post-hoc observed power calculation was performed, as effect estimates and their 95% confidence intervals were considered more informative for interpreting the precision of the findings.

## Results

A total of 53 root canal treatments in 48 subjects (mean age 45.4 ± 12.1 years) were included in the analysis. Baseline patient characteristics are reported in Table 3. Among these, 29 canals were obturated using the single cone technique and 24 with a carrier-based technique. Tooth-related baseline characteristics of the two obturation groups are reported in Table 4.

**Table 4:** Teeth related characteristics of the 2 obturation groups

Tooth level	Single cone + Neosealer Flo *n* = 29	Carrier-based + Neosealer Flo *n* = 24	Chi-square
*Tooth type*			
Anterior	5 (17.3)	7 (29.2)	Chi = 1.16
Premolar	6 (20.7)	5 (20.8)	*p* = 0.559
Molar	18 (62)	12 (50)	
*Tooth location*			
Maxilla	16 (55.2)	10 (41.6)	Chi = 0.96
Mandible	13 (44.8)	14 (58.4)	*p* = 0.327
*Diagnosis*			
Prosthetic reasons	1 (3.4)	4 (16.7)	Chi = 5.49
Pulpitis	8 (27.6)	3 (12.5)	*p* = 0.138
Pulp necrosis	10 (34.5)	12 (50)	
Exacerbated periapical lesion	10 (34.5)	5 (20.8)	
*Apical diameter*			
25–40	24 (82.8)	17 (70.8)	Chi = 1.06
45–60	5 (17.2)	7 (29.2)	*p* = 0.301
*Obturation quality*			
underfilling	6 (20.7)	3 (12.5)	Chi = 3.66
adequate	10 (34.5)	4 (16.7)	*p* = 0.159
overfilling	13 (44.8)	17 (70.8)	
*Diameter of extrusion*,* mm*			
0	15 (51.8)	6 (25)	Chi = 12.37
0.1–1.0	4 (13.8)	1 (4.1)	*p* = 0.014
1.1–2.0	2 (6.8)	7 (29.2)	
2.1–5.0 > 5.1	8 (27.6) 0 (0)	6 (25) 4 (16.7)	
*Sealer’s resorption*			
Yes	3 (10.3)	2 (8.3)	Chi = 4.559
Partially	5 (17.2)	7 (29.2)	*p* = 0.207
No	6 (20.7)	9 (37.5)	
No extrusion	15 (51.8)	6 (25)	
*Definitive restoration*			
Composite	16 (55.2)	17 (70.8)	Chi = 1.57
Crown	10 (34.5)	6 (25)	*p* = 0.455
Post	3 (10.3	1 (4.1)	
*Initial PAI*			
No lesion (PAI 1)	7 (24.2)	4 (16.7)	Chi = 1.07
Enlarged periapical space (PAI 2)	9 (31)	6 (25)	*p* = 0.785
Initial periapical lesion (PAI 3)	4 (13.8)	5 (20.8)	
Periapical lesion (PAI 4)	9 (31)	9 (37.5)	

The 36-month outcome according to tooth-related characteristics is reported in Table 5. Healing was observed in 27/29 teeth in the single-cone group (93%; 95% CI 78.0%–98.1%) and in 21/24 teeth in the carrier-based group (87.5%; 95% CI 69.0%–95.7%). The absolute difference in healing rates between groups was 5.6% (95% CI −10.5% to 21.7%). There was no statistically significant difference between the two techniques in terms of healing outcome (*P* > 0.05). Complications/events during the follow-up are summarized in Table 6.

**Table 5:** 36-month outcome in the two obturation groups according to tooth related characteristics

Tooth level	Single cone + Neosealer Flo Healed *n* = 27	Carrier-based + Neosealer Flo Healed *n* = 21
*Tooth type*		
Anterior	5 (18.5)	7 (33.3)
Premolar	6 (22.2)	5 (23.9)
Molar	16 (59.3)	9 (42.8)
*Tooth location*		
Maxilla	15 (55.5)	9 (42.8)
Mandible	12 (44.5)	12 (57.2)
*Diagnosis*		
Prosthetic reasons	1 (3.7)	4 (19.1)
Pulpitis	8 (29.6)	3 (14.3)
Pulp necrosis	9 (33.3)	11 (52.4)
Exacerbated periapical lesion	9 (33.3)	3 (14.3)
*Apical diameter*		
25–40	22 (81.5)	15 (71.4)
45–60	5 (18.5)	6 (28.6)
*Obturation quality*		
underfilling	6 (22.2)	3 (14.3)
adequate	10 (37)	3 (14.3)
overfilling	11 (40.8)	15 (71.4)
*Diameter of extrusion*,* mm*		
0	15 (55.5)	6 (28.7)
0.1–1.0	4 (14.8)	1 (4.7)
1.1–2.0	2 (7.5)	7 (33.3)
2.1–5.0	6 (22.2)	5 (23.8)
> 5.1	0 (0)	2 (9.5)
*Sealer’s resorption*		
Yes	3 (11.2)	2 (9.5)
Partially	3 (11.1)	7 (33.3)
No	6 (22.2)	6 (28.6)
No extrusion	15 (55.5)	6 (28.6)
*Definitive restoration*		
Composite	14 (51.9)	15 (71.4)
Crown	10 (37)	5 (23.8)
Post	3 (11.1)	1 (4.8)
*Initial PAI*		
No lesion (PAI 1)	7 (25.9)	4 (19)
Enlarged periapical space (PAI 2)	9 (33.4)	6 (28.6)
Initial periapical lesion (PAI 3)	4 (14.8)	4 (19)
Periapical lesion (PAI 4)	7 (25.9)	7 (33.4)

**Table 6:** Complications/events occurred during the follow-up

	*n*	Healed	Periapical lesion	Extracted
Carrier-based + Neosealer Flo	24	21 (87.5)	1 (4.2)	2 (8.3)
Single cone + Neosealer Flo	29	27 (93)	1 (3.5)	1 (3.5)

Apical sealer extrusion was more frequently observed in the carrier-based group, with 18 out of 24 teeth (75%) showing extrusion, compared to 14 out of 29 teeth (48.2%) in the single cone group. Representative radiographic cases of both obturation techniques and extrusion patterns are shown in Fig. 2. Mean extrusion size was greater in the carrier-based group, and significantly higher extrusion (in millimeters) was associated with both the use of carrier-based obturation and with cases presenting a preoperative PAI score greater than 3. Extrusion size comparisons according to initial PAI, obturation technique and final apical diameter are reported in Table 7. Extrusion size comparisons are illustrated in Fig. 3.

**Table 7:** Extrusion comparisons according to initial PAI, obturation technique and final apical diameter. Different small letters indicate a statistically significant difference between parameters (*p*<0.050)

Periapical Index (PAI)	*n*	Diameter of extrusion (Mean ± SD)	Median [IQR]	Confidence interval
PAI 1–2	26	0.8 ± 1.3 a	0 [0 - 1.3]	95% IC 0.5 −1.1
PAI 3–5	27	2.1 ± 1.9 b	1.9 [1.1–2.9]	95% IC 1.6 - 2.7
Obturation technique				
Single cone + Neosealer Flo	29	1.1 ± 1.4 a	0 [0–2.0]	95% IC 0.7 −1.5
Carrier-based + Neosealer Flo	24	2.0 ± 2.0 b	1.5 [0.2–2.6]	95% IC 1.5 −2.6
Final apical diameter				
*25–40*	41	1.37 ± 1.52 a	1.01 [0–1.8]	95% IC 0.94 - 1.79
*45–60*	12	1.95 ± 2.41 a	1.21 [0–2.6]	95% IC 1.29 - 2.61

Radiographic modification of extruded sealer over time was noted in 5 of the 53 cases (9.4%), regardless of the obturation technique used. These cases were distributed across both techniques (3 cases in the single-cone group and 2 cases in the carrier-based group), whereas the majority of extrusions remained radiographically stable over time (Table 8**).** These results suggest that both obturation methods are associated with high long-term healing success, despite differences in the frequency and extent of apical extrusion.

**Table 8:** Extrusions events occurred between the groups

Sealer resorption	*N*	Stable	Resorbed	No extrusion
Single cone + Neosealer Flo	29	11 (37.9)	3 (10.3)	15 (51.7)
Carrier-based + Neosealer Flo	24	16 (66.7)	2 (8.3)	6 (25.0)

## Discussion

This retrospective study evaluated the clinical and radiographic outcomes of root canal treatments performed using a premixed calcium silicate–based sealer in combination with either a cold single-cone technique or a carrier-based obturation protocol. After a minimum follow-up of 36 months, both approaches demonstrated high periapical healing rates (93% and 88%, respectively), with no statistically significant differences observed between the two techniques. Within the limitations of the present study, the first null hypothesis could not be rejected, as comparable clinical success and periapical healing were achieved irrespective of the obturation technique adopted. These findings indicate that, when NeoSealer Flo is used, its clinical effectiveness in supporting long-term periapical healing is not adversely influenced by the obturation technique. Consistent with the comparable clinical outcomes observed between the two obturation techniques, these results confirm previous findings in which premixed calcium silicate–based sealers achieved high success rates when used with both warm and cold obturation techniques in follow-up periods, such as 88.6% at 24 months when using carrier-based technique and Ceraseal Premixed sealer, 85.4% at 3 years using a single cone technique and Ceraseal premixed sealer, 90.9% at 3 years using single cone technique and Endosequence premixed sealer or 89.6% at 4 years using a single cone technique and Bioroot RCS calcium silicate sealer [26, 31–34]. The present investigation extends the available evidence to a longer 36-month follow-up and further supports the concept that bioactive sealers may partially compensate for reduced gutta-percha compaction associated with cold techniques due to their ability to release calcium ions, stimulate mineral deposition, and establish a favorable biological interface.

Previous clinical and laboratory investigations on NeoSealer Flo have reported favorable biological properties, despite some variability in physicochemical parameters such as setting kinetics and flowability [35, 36]. Calcium silicate–based sealers have consistently demonstrated good biocompatibility and immunomodulatory effects compared with resin-based materials, a behavior largely attributed to their alkalizing activity and sustained calcium ion release [37]. From a biological perspective, these characteristics have been associated with a reduced early inflammatory response, as supported by experimental and clinical evidence indicating lower neuroinflammatory activation, oxidative stress, and postoperative discomfort when calcium silicate–based sealers are used [38]. Recent randomized clinical trials and systematic reviews have further shown that, although premixed bioceramic sealers such as NeoSealer Flo may exhibit a higher incidence of sealer extrusion due to their flowable nature, this does not necessarily translate into worse clinical outcomes or increased postoperative discomfort when compared with epoxy resin–based sealers [4, 39]. Taken together, these material-related characteristics may help explain the high rates of periapical healing observed in the present study, independently of the obturation technique employed. In addition, bioceramic sealers have shown antimicrobial activity, which is considered a desirable property in endodontic materials as it may contribute to the control of residual intraradicular infection. *Enterococcus faecalis*, a facultative anaerobic Gram-positive bacterium frequently associated with persistent endodontic infections and biofilm formation, has been reported to be susceptible to freshly mixed calcium silicate–based sealers [40]. This effect appears to be more pronounced during the early setting phase and has been attributed to the formation of calcium hydroxide during the hydration reaction, resulting in an alkalizing environment capable of disrupting bacterial membranes and genetic material [36, 41].

Notably, despite in vitro concerns regarding the potential influence of heat on the setting behavior and physicochemical stability of calcium silicate–based sealers [42], the present clinical findings did not reveal any detrimental effect associated with the use of carrier-based obturation techniques. It was reported sealers dehydration and degradation of organic components (such as PEG) upon heating the materials at high temperatures (100–225 °C degrees), thereby affecting some physical properties, such as setting time, flowability [43–45] and film thickness [43–45]. Thermafil is typically heated to 60 °C in the dedicated Thermaprep oven. However, this technique hardly reaches such temperature due to the rapid dissipation of temperatures when placed inside the canals [46]. A previous study showed that the real-time temperature within the root canal is submitted to only a negligible increase in temperature (< 1 °C around the tooth) [46]. A previous study demonstrated that heat application of from 37 °C up to 77 °C are not sufficient to induce modifications of flow into the root canal, neither can modify the setting of the material [47]. This suggests that the biological and clinical performance of NeoSealer Flo may remain stable under clinical thermal conditions, with any temperature-related effects likely being transient and without measurable impact on medium- to long-term periapical healing.

The combination of bioactivity, favorable tissue response, and early antimicrobial potential may help explain the limited postoperative discomfort and the high rates of periapical healing observed in the present study, supporting the clinical effectiveness of NeoSealer Flo over the medium to long term, independently of the obturation technique employed. While clinical effectiveness appeared independent of the obturation technique, differences in material behavior (particularly in terms of sealer extrusion) require specific consideration. Accordingly, the second null hypothesis was partially rejected, as the obturation technique influenced the frequency and extent of sealer extrusion, whereas radiographic modification of the extruded material did not appear to be technique-specific in this cohort.

As expected, sealer extrusion occurred more frequently and with greater volumes in the carrier-based filling group, particularly in teeth presenting with pre-existing apical lesions (PAI > 2). This finding is consistent with existing literature reporting higher extrusion rates associated with thermoplastic techniques, likely related to heat-induced sealer expansion and increased hydraulic pressure during carrier insertion [4]. Despite these differences, periapical healing rates remained high, and no statistically significant association was observed between sealer extrusion and treatment failure. This observation agrees with ex vivo studies showing that extruded calcium silicate sealers induce a limited inflammatory response [48] and may undergo progressive encapsulation or partial resorption over the time [49]. In the present study, only a small proportion of cases (9.4%) exhibited radiographic modification of the extruded material during follow-up, suggesting that majority of extrusions remained radiographically stable and clinically asymptomatic.

Notably, extrusion size was associated with both the use of warm obturation techniques and higher preoperative PAI scores. Severe periapical inflammation may result in widening of the apical foramen, thereby facilitating material extrusion [50]. These findings suggest that preoperative lesion characteristics, rather than obturation technique alone, play a relevant role in influencing extrusion behavior. The effect of apical sealer extrusion on healing outcome remains debated. Previous studies suggested that extrusion may negatively influence healing when associated with overextension and incomplete three-dimensional obturation, or when occurring in teeth with pre-existing apical periodontitis [51–53]. In such cases, the unfavorable outcome may depend on inadequate apical sealing, but also on the quantity and extruded sealer composition. Some sealers, such as zinc oxide-eugenol based could exert irritation, cytotoxicity or foreign-body reaction due to the presence of pro-inflammatory compounds (as eugenol) [54].

In the present study, sealer extrusion was frequent, particularly in the carrier-based group and in teeth with preoperative PAI 3–5, yet healing rates remained high and no clear detrimental association with treatment failure emerged. This finding may be partially explained by the specific biological behavior of calcium silicate–based sealers, whose solubility, calcium ion release, and bioactivity may promote apatite nucleation and a more favorable tissue response compared with traditional sealers. Extruded material may undergo radiographic modifications or complete resorption. This was speculated for different reasons as solubilization, phagocytosis, or fibrous encapsulation [55]. The size and morphology of extrusion also appear to be influenced by local periapical conditions, since apical bone defects may facilitate larger and more circular extrusion patterns, whereas apical bone integrity may limit material spread beyond the foramen.

The findings of this study contribute important practical insights. Increased extrusion in warm techniques did not compromise healing, reducing concerns regarding the biological consequences of extrusion when using calcium silicate sealers. Radiographic stability of extruded material was the most frequent event, with few cases showing modification over time. Moreover, operator experience, represented in this study by postgraduate operators, seems not to negatively influence treatment outcomes, as no intraoperative complications (instrument fractures, perforations or missed canals) were observed. The use of techniques with a rapid learning curve (i.e. single cone techniques and carrier-based techniques) and standardized protocols were specifically conceived to be used in such university context.

Nevertheless, the retrospective design, heterogeneous case selection, and limited sample size represent important limitations of the present study. The low number of unfavorable healing events, which limited the possibility of performing reliable multivariable analyses and reduced the ability of the study to detect small between-group differences. Future prospective and preferably randomized clinical trials are warranted to confirm these findings and to further investigate the influence of temperature on setting kinetics, interfacial adaptation, and long-term behavior of bioceramic sealers under different obturation conditions.

## Conclusion

Both single cone and carrier-based obturation techniques, when used with a premixed calcium silicate sealer, achieved high healing rates at 36 months. Although extrusion was more frequent and extensive in warm techniques, it did not affect the clinical outcome. These results support the flexible use of NeoSealer Flo with either obturation method and provide reassurance regarding the biological behavior of apically extruded bioceramic sealer.

## Data Availability

Data available upon reasonable request.
